# A Comparison of Two Short-Course Primaquine Regimens for the Treatment and Radical Cure of *Plasmodium vivax* Malaria in Thailand

**DOI:** 10.4269/ajtmh.2010.09-0428

**Published:** 2010-04

**Authors:** Sasithon Pukrittayakamee, Mallika Imwong, Kesinee Chotivanich, Pratap Singhasivanon, Nicholas P. J. Day, Nicholas J. White

**Affiliations:** Department of Clinical Tropical Medicine, Faculty of Tropical Medicine, Mahidol University, Bangkok, Thailand; The Royal Institute, Grand Palace, Bangkok, Thailand; Centre for Tropical Medicine, Nuffield Department of Clinical Medicine, Churchill Hospital, Oxford, United Kingdom

## Abstract

Thai adult males (*N =* 85) with acute *Plasmodium vivax* malaria and normal glucose-6-phosphate dehydrogenase screening were randomized to receive 30 mg or 60 mg primaquine daily for 7 days (*N* = 43 and 42, respectively). The regimens were well tolerated and all patients recovered fully. Median fever clearance (47 hours; range 4 to 130 hours), mean ± SD parasite clearance times (87.7 ± 25.3 hours), gametocyte clearance, and adverse effects were similar in the 2 groups. Two patients, 1 from each group, had a 30% reduction in hematocrit. The cumulative 28 day relapse rate (95% confidence interval) by Kaplan Meier survival analysis was 29% (16–49%) in the 30 mg group compared with 7% (2–24%) in the 60 mg group; *P* = 0.027. Comparison with previous data obtained at this same site suggests that the recurrences comprised approximately 17% recrudescences and 12% relapses in the 30 mg/day group compared with 3% recrudescences and 4% relapses in the 60 mg/day group. These data suggest that the dose-response relationships for primaquine's asexual stage and hypnozoitocidal activities in-vivo are different. A 1 week course of primaquine 60 mg daily is an effective treatment of vivax malaria in this region.

## Introduction

Primaquine is the only widely available hypnozoitocidal drug for *Plasmodium vivax* and *P. ovale* malaria. Primaquine at the dose most usually recommended in the past for radical cure (15 mg base adult dose daily for 14 days) is also weakly effective against asexual stages of *Plasmodium vivax* malaria.^1^ The standard chloroquine and primaquine treatment regimen may therefore be considered as a combination treatment. The dose-response relationship for primaquine's asexual stage activity is uncertain. Higher doses of primaquine may be more effective than standard doses but also increase the risk of adverse effects. Primaquine is associated with upper gastrointestinal discomfort, particularly if taken on an empty stomach, and causes oxidant hemolysis in subjects who have glucose-6-phosphate dehydrogenase (G6PD) deficiency. In malaria prophylaxis, a 30 mg daily dose of primaquine has been shown to be well tolerated even when given continuously for as long as 20 weeks.^2^,^3^ For radical cure of vivax malaria in normal G6PD patients, adult doses of primaquine at 22.5 to 30 mg proved safe and effective in Thailand,^4^,^5^ and the higher dose (0.5 mg base/kg) has recently been recommended by the World Health Organization. In Thailand *Plasmodium vivax* has a 3 week interval between primary infection and the first relapse, and approximately half the patients experience at least 1 relapse.^5^–^8^^-^ The schizontocidal activity of primaquine^1^ becomes increasingly important in the face of increasing chloroquine resistance in *P. vivax*, although the 2 week course currently recommended for radical cure remains a significant impediment to good adherence and therefore optimal efficacy. The dose-response relationships for primaquine's asexual stage and hypnozoitocidal activities have not been well characterized. To compare these 2 discrete activities and to assess efficacy and tolerability, we evaluated 7-day courses of 30 mg and 60 mg daily primaquine-only regimens for the treatment and radical cure of *Plasmodium vivax* malaria in Thailand.

## Patients and Methods

### Patients.

The study was conducted in adult male patients with acute symptomatic *Plasmodium vivax* malaria admitted to the Bangkok Hospital for Tropical Diseases, Thailand between 1996 and 1998. The study was approved by the ethics committee of the Faculty of Tropical Medicine, Mahidol University, Bangkok. All patients gave full informed consent.

All patients were screened and only those with normal G6PD testing were included. Other exclusion criteria were a history of drug hypersensitivity, having taken any antimalarial drugs within the previous 48 hours, or urine screening tests that were positive for sulfonamides (lignin test) or 4-aminoquinolines (Wilson Edeson test). Patients who had patent mixed infections or were unable to stay in the hospital until clearance of both fever and parasitemia were excluded from the study.

### Management.

After clinical assessment and confirmation of the diagnosis from thick and thin blood smears, baseline blood samples were taken for routine hematology and biochemistry. Treatments were allocated according to double-blinded randomization codes. The treatment allocation was kept in serially numbered sealed envelopes that were opened only after the patient had been enrolled in the study. Patients were randomized to receive 1 of the following 2 regimens:

1. Primaquine (Thai Government Pharmaceutical Organization) 0.50 mg base/kg once daily (adult dose 30 mg base/day) for 7 days
2. Primaquine 1.0 mg base/kg once daily (adult dose 60 mg base/day) for 7 days.

Oral acetaminophen (0.5–1 g 4 hourly) was given for fever > 38°C.

### Assessments of clinical response.

Vital signs were recorded every 4 hours until resolution of fever and thereafter every 6–12 hours. Fever clearance time was defined as the time for body temperature to fall below 37.5°C and remain below this value for > 48 hours. Early treatment failure was defined as persistence of fever and parasitemia for > 7 days or persistence of parasitemia in the absence of fever for > 14 days. Reappearance of infection was assessed in patients who remained in Bangkok either in the hospital or at home (i.e., outside the malaria transmission area) for at least 28 days. Patients who failed to respond to the treatment, or those who had recurrent vivax infections were treated subsequently with the standard regimen of chloroquine (25 mg/kg base over 3 days) and primaquine (0.25 mg base/kg/day for 14 days). Patients with delayed appearance of *P. falciparum* infection were treated with a 7-day course of quinine (10 mg salt/kg every 8 hours) combined with tetracycline (250 mg every 6 hours) and censored from that time in the subsequent assessment.

Assessment of gastrointestinal symptoms (nausea, vomiting, diarrhea, and abdominal pain) and any other symptoms and signs was performed every day during and after primaquine treatment (day 0 to day 7, day 14, and day 28). Hematocrit was assessed daily during and after primaquine treatment. Laboratory investigations for full blood count and routine biochemical tests were performed before treatment (D0) and then on day 7, day 14, and day 28.

### Parasitological assessment.

Parasite counts of both sexual and asexual stages were measured every 2–12 hours in thin films and thick films until the parasitemia became detectable only in thick films, then every 12 hours until clearance, and thereafter daily for 28 days. Parasitemia was expressed as the number of parasites per microliter of blood (derived from the numbers of parasites per 1,000 red blood cells in the thin film stained with Giemsa or Field's stain or calculated from the white count and the numbers of parasites per 200 white blood cells in the thick film). Gametocytes and asexual stages were counted separately. Gametocyte counts were calculated from the thick film only. Gametocyte carriage was expressed as the proportion of patients with patent gametocytemia either before treatment (i.e., on admission) or at any time after the start of treatment. The gametocyte clearance time was defined as the interval from the first detection of gametocytes in peripheral blood films either before or after treatment to the last detection of gametocytes. The variables describing parasite clearance were time taken until the asexual malaria parasite count fell by 50% (PC50), time taken to fall by 90% (PC90) of the pre-treatment admission value, and the time taken to fall below detectable levels in a peripheral blood smear (PCT); parasite reduction ratio (PRR) was the ratio of the parasite count before treatment to the counts at 24 hours and at 48 hours. Recurrence of vivax malaria could have resulted from recrudescence or relapse but not re-infection because these patients remained out of the transmission area throughout the follow-up period. In an individual patient whose parasitemia cleared, recrudescence or relapse of *P. vivax* cannot be distinguished reliably.^9^ Reappearance of parasitemia within 28 days was therefore considered together as treatment failure. Reappearance rates were also compared with other studies conducted in the same hospital with similar patient populations, similar study procedures, and similar follow-up in which the dose and duration of primaquine treatment varied.^5^,^7^,^8^

### Statistical analysis.

The data from each treatment group were compared by one-way analysis of variance. Non-parametric data were compared by the Kruskal-Wallis test. The cumulative cure rates were calculated by Kaplan-Meier survival analysis censoring for loss to follow-up or appearance of *P. falciparum* and were compared using the log rank test. The Spearman rank correlation coefficient was used to evaluate the association between gametocyte counts, parasite counts, fever clearance, and parasite clearance times. All statistical analyses were performed using the statistical computing package SPSS Version 16.0 for Windows (SSPS Inc, Chicago, IL).

## Results

### Patients.

There were 85 patients with acute vivax malaria 14–61 years of age (mean ± SD = 24.6 ± 10.1 years). The majority of patients acquired malaria infection from the western border of Thailand (*N* = 74, 87.1%) and a high proportion (*N* = 37; 43.5%) had had at least one malaria infection in the past year (median 2; range 1 to 10 attacks). All patients presented with a history of fever (median 4; range 1 to 20 days) before hospital admission. The geometric mean (range) peripheral blood *P. vivax* asexual parasite count was 6,205/µL (33 to 97,968/µL). Overall 29% (*N =* 25) of patients had detectable gametocytemia at presentation. The median (range) gametocyte count was 41/µL (16–110/µL). The majority of patients (88%) were thrombocytopenic (platelets < 180,000/µL) but none had significant abnormalities of other baseline hematology results (Table 1). Between the two treatment groups randomized to receive 30 mg or 60 mg primaquine daily for 7 days (*N* = 43 and 42), serum glutamic-pyruvic transferase (sGPT) concentrations were significantly higher in the high dose group (median 24; range 8 to 84 versus 19; 5 to 147 U/liter, *P* = 0.045) but all baseline characteristics and other laboratory findings were similar in the 2 groups (*P* ≥ 0.24).

### Clinical course.

**Figure 1.**
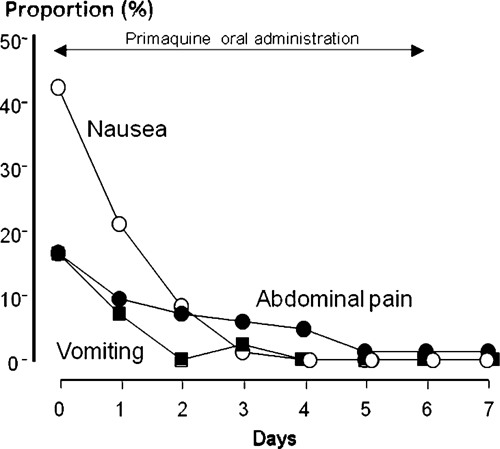
Proportions of patients with gastrointestinal symptoms during primaquine monotherapy for *P. vivax* malaria.

All patients made a full clinical recovery after treatment. One patient developed acute tonsillitis on day 5 but responded well to antibiotics (amoxicillin). His fever cleared 201 hours after starting primaquine treatment. The overall mean (SD) parasite clearance time was 87.7 hours (25.3 hours). The overall median (range) parasite reduction ratios at 24 hours and 48 hours were 2 (0.1–4.1) and 14.4 (0.1–1376), respectively. Between the 2 treatment groups, there were no significant differences in PCT or parasite reduction ratios (*P* ≥ 0.18). Excluding the patient with acute tonsillitis, the median fever clearance time was 47 hours (range 4 to 130 hours). On admission, 55 patients (64.7%) had gastrointestinal symptoms i.e. nausea (*N* = 36, 42.4%), vomiting (*N =* 14, 16.5%), abdominal pain (*N =* 14, 16.5%), and diarrhea (*N =* 3, 3.5%). These symptoms disappeared after day 3 in the majority of patients (*N =* 48/55; 87.3%) as the malaria infection subsided (Figure 1). Only one patient who received daily 60 mg primaquine had persistent but mild abdominal pain till day 7 but this responded well to antacids. His pain resolved fully on the eighth day suggesting it was drug related. Transient localized rashes developed in 3 patients on day 0 to day 3 but all lasted only for 24 hours. Comparing the 2 treatment groups, the overall incidence of gastrointestinal symptoms during acute malaria (day 0–3) was significantly higher in the lower dose primaquine group (33/43 versus 22/44; *P* = 0.033). There was no other significant adverse effects or complications in the studied patients.

### Laboratory results.

**Figure 2.**
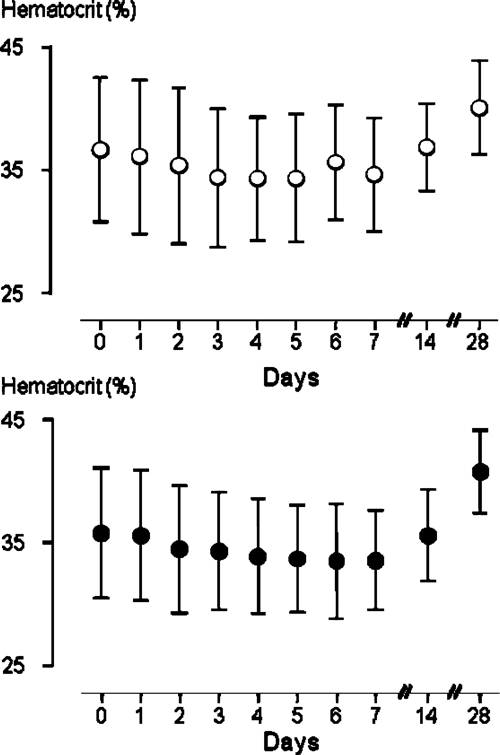
Serial hematocrits (%) levels in adult G6PD normal patients treated with primaquine of 30 mg/day (open circles) or 60 mg/day (closed circles) for acute vivax malaria. Data are shown as mean (SD).

The mean ± SD baseline hematocrit of all patients (36.3% ± 5.6%) decreased significantly after day 2 to day 7 (Figure 2, *P* < 0.001), then returned back to baseline values on day 14 (36.2% ± 3.7%; *P =* 0.07), and became significantly higher than the baseline hematocrit on day 28 (40.5% ± 3.6%, *P =* 0.001). The overall median hematocrit reduction was 8.8% (range 2.2–33.3%). A hematocrit drop of > 30% was observed in 2 cases; one from each group. The lowest recorded value was 30% on day 6 in one case and 28% on day 7 for the other. Neither was associated with symptoms and none of the studied patients needed a blood transfusion. Comparing the 2 treatment groups, there were no significant differences in the baseline hematocrit values (36.7 ± 5.9% versus 35.9 ± 5.3%; *P* = 0.50), the proportion of patients with hematocrit reduction (*N* = 40/43 versus 37/42; *P* = 0.34) or the magnitude of hematocrit reductions (Figure 2, *P* ≥ 0.11).

On admission, thrombocytopenia (platelets < 180,000/µL) was found in the majority of patients (87.8%; *N =* 72/82). The mean ± SD baseline platelet count was 111,000 ± 77,000/μL but then increased to above 200,000/μL in all subsequent follow-up assessments (day 7, day 14, and day 28) in both groups (Table 2). Except for the reduction in hematocrit and thrombocytopenia on admission, none of the studied patients showed significant persistent abnormal hematology findings during 1 month monitoring. An elevation of bilirubin > 3 mg/dL was found on admission only in 6 patients (4 group 1, 2 group 2) and in 2 was accompanied by SGPT elevation (52 and 64 Units/L). During the follow-up assessments, serum SGPT elevation of > 2-fold was found in 8 patients but all were transient and mild (maximum range = 84 to 199 Units/L). Except for one patient with a serum creatinine of 2.2 mg/dL on admission only, none of the studied patients had significant elevation of serum creatinine (> 2.0 mg/dL) throughout the follow-up period.

### *Plasmodium vivax* gametocytemia.

During hospital stay, patent gametocytemia was detected in 62.4% (*N* = 53) of patients, 29.4% (*N =* 25) on admission and a further 32.9% (*N =* 28) after starting primaquine treatment. The gametocyte carriage of all studied patients (i.e., including those without gametocytemia) was brief; median was 12 hours (range, 0–74 hours) and was not significantly different between the two treatment groups (median 12; range 0–74 versus 7; 0–60 hours; *P* = 0.49). The overall gametocyte carriage rates were not significantly different between the 2 groups either before (*N* = 15 and 10) or after treatment (*N* = 14 and 14) (*P* = 0.58).

Gametocytemia before treatment and the ratio to asexual stage parasite densities were similar in the two treatment groups (Table 3). In patients with post-treatment gametocytemia, the 60 mg primaquine group showed slightly better suppression of gametocytemia compared with the 30 mg primaquine group with lower peak gametocyte/corresponding asexual parasite counts ratios (0.6% versus 3%, *P* = 0.016).

### Recurrence rates.

Complete clinical and parasitological follow-up was obtained in 64 patients (75%). These patients were either followed-up outside the malaria transmission area for 28 days or remained in the hospital until the subsequent appearance of vivax or falciparum malaria. The remaining 25% of patients did not return after discharge. Their demographic and disease characteristics were similar to those successfully followed. There were no significant differences in proportion of loss to follow-up between the two treatment groups (12/43; 27.9% in the 30 mg groups versus 9/42; 21.4% in the 60 mg groups, *P* = 0.62).

**Figure 3.**
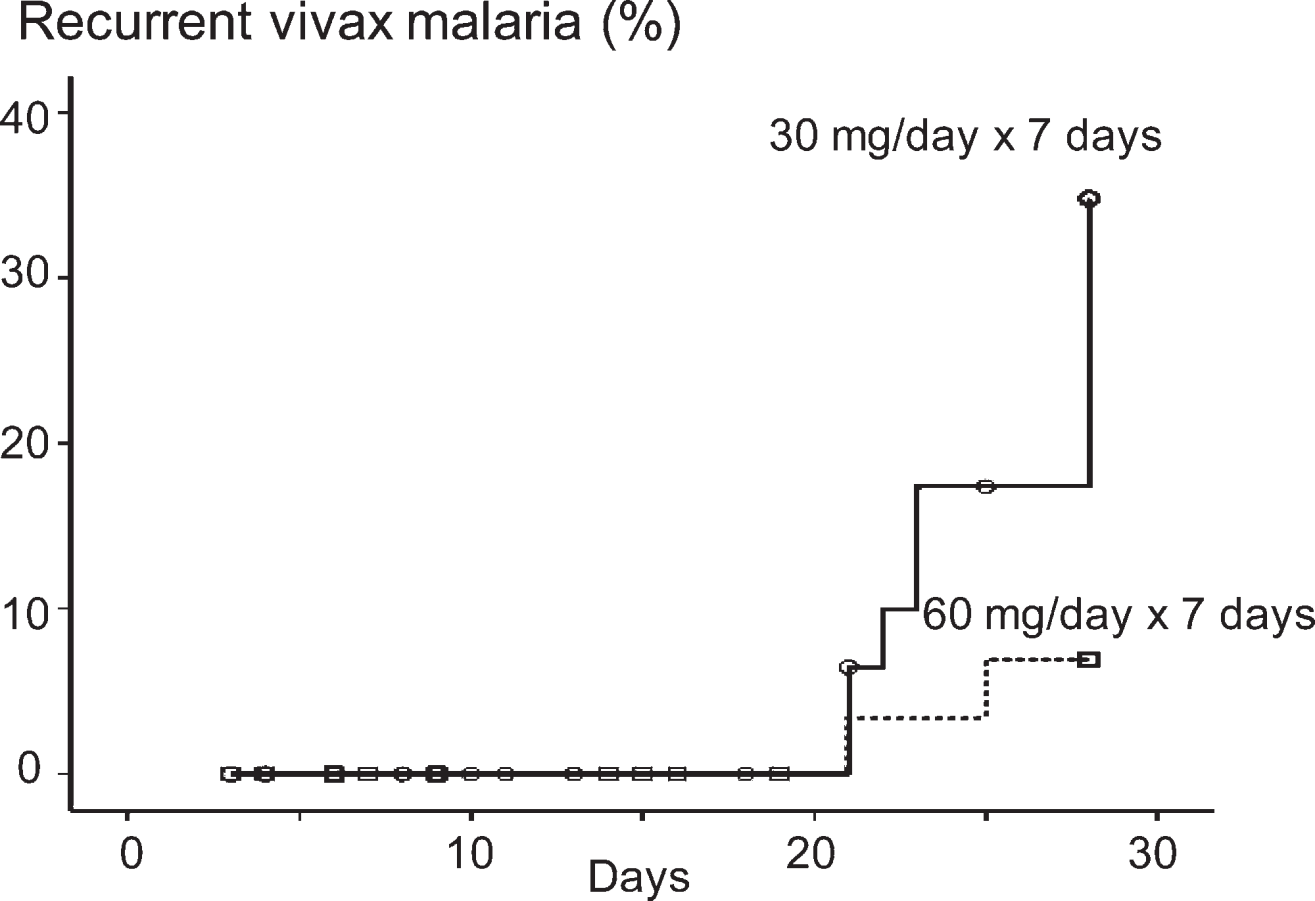
Proportions of adult patients with recurrent *P. vivax* parasitemia in the 30 mg/day and 60 mg/day primaquine groups.

Of the 31 patients successfully followed in the 30 mg group, 9 (29%) had reappearance of vivax and another one (3%) had emergence of falciparum malaria after a full clinical recovery and parasite clearance of the primary vivax illness. The corresponding figures were 2/33 (6%) relapses and 3 (7%) *Plasmodium falciparum* recrudescences in the 60 mg group. The incidence of vivax reappearance was significantly lower in the high dose primaquine group; by Kaplan Meier survival analysis with day 28 relapse estimates (95% Confidence interval) of 29% (16–49%) in the 30 mg group compared with 7% (2–24%) in the 60 mg group; *P* = 0.027 (Figure 3).

**Figure 4.**
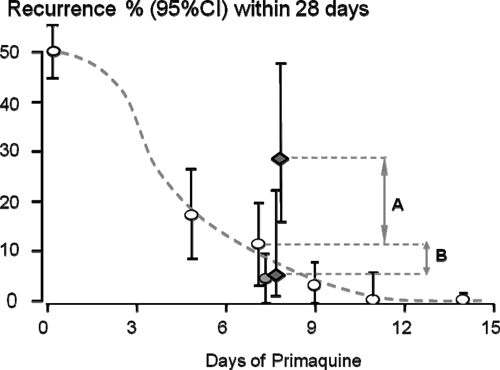
Recurrences following acute *Plasmodium vivax* malaria in adults studied in Thailand in a hospital setting for 28 days where reinfection was excluded. The horizontal axis shows the number of days for which primaquine was given. The open circles and bars represent the proportion and its 95% confidence interval of recurrences in patients who also received artesunate for 5 or 7 days, which has no hypnozoitocidal activity but is highly effective against the blood stage infection, together with primaquine at a daily adult dose of 30 mg (base).^7^,^8^ These recurrences are therefore assumed to be relapses. The grey circle shows the recurrences in adults receiving artesunate and primaquine 60 mg daily (7 days).^7^ The black diamonds are the results of the present study in which primaquine was given alone. The difference between the primaquine 30 mg/day with and without artesunate reflects recrudescences (**A**; arrowed) and that between primaquine 30 mg/day with artesunate and the primaquine 60 mg/day regimens presumably reflects relapses (**B**; arrowed). See Supplemental Appendix. (Raw data compiled from the present study and references^7^,^8^).

All patients with incomplete cure responded to retreatment with the corresponding standard regimens for vivax malaria or falciparum malaria. To assess these responses with primaquine monotherapy, we compared the results of the present study with the previously reported studies conducted in the same hospital (so reinfection could be excluded) and with similar patient groups with acute vivax malaria, in which primaquine (30 mg base or 60 mg base per day) was given together with artesunate (5 or 7 days) for 3, 5, or 7 days.^5^,^7^,^8^ Artesunate has no hypnozoitocidal activity but is highly effective against the blood stage infection. Recurrences after artesunate regimens are therefore assumed to be relapses (see Supplemental Appendix, available at www.ajtmh.org). The 60 mg/day recurrence rates in this study are similar to those in the previous studies, which suggests first that this dose gives nearly maximal hypnozoitocidal activity, and second that the significantly higher recurrence rate with the 30 mg/day primaquine monotherapy results predominantly from recrudescences (Supplemental Appendix and Figure 4).

### Factors associated with gametocyte carriage.

The admission asexual parasite counts were not significantly different between patients with or without patent gametocytemia (Table 4) that appeared either before or after treatment (*P* ≥ 0.11). Regardless of treatment groups, patients with gametocytemia cleared asexual parasite much slower that those without gametocytemia (*P* ≤ 0.018) and also had slower fever clearance times although this was not statistically significant (*P* = 0.075). This was not attributable to higher parasitemia as there were no significant differences in parasitemia between those with and without gametocytemia. There were also no significant differences in the numbers of previous malaria attacks, duration of fever, or admission hematocrit values or reappearance rate of vivax malaria between patients with and without gametocytemia (Table 4).

## Discussion

Primaquine may be the most under-used antimalarial drug. Although widely recommended for the radical cure of relapsing malarias (*P. vivax, P. ovale*), it is seldom actually prescribed in endemic areas in the 14 day courses currently considered necessary for optimum effects.^10^,^11^ Lack of availability of testing for glucose-6-phosphate dehydrogenase, concerns over the potential for severe hemolysis, and poor adherence to long courses conspire to prevent widespread use. As *P. vivax* is traditionally considered more difficult to eliminate than *P. falciparum*, current global initiatives against vivax malaria may be unsuccessful if primaquine remains only a theoretical recommendation. Chloroquine resistance in *P. vivax* is now established in several areas, notably Oceania and in Indonesia. As primaquine has significant asexual stage activity against *P. vivax*, the combination of chloroquine and primaquine would be expected to delay or prevent the emergence of resistance. India, which harbors the majority of the world's *P. vivax* infections, recommended an inadequate primaquine dose regimen (5 days only) for radical cure for nearly half a century, but perhaps the reason that chloroquine resistance did not emerge there was because of combination treatment against the asexual stage infection. If an effective radical cure could be given in a shorter duration than 2 weeks, then adherence would be expected to improve.

Chloroquine is still the most widely used vivax malaria treatment in the world. Given alone for tropical frequent relapse *P. vivax* infections, the first relapse which usually emerges at 3 weeks in Southeast Asia, is suppressed by residual therapeutic levels of chloroquine in the blood (post treatment prophylaxis). Assessment of radical curative regimens with chloroquine therefore require long follow-up, and may underestimate true relapse rates. It is not known whether the suppressed relapse is prevented or delayed. The 8-aminoquinolines are the only class of drug with radical curative activity, although there is intriguing evidence that other quinoline antimalarials may synergize with them in this activity.^12^ Soon after the introduction of primaquine over 50 years ago it was established that radical cure was a function of the total dose given. Daily dosing was limited by toxicity. In this study of G6PD normal Thai adult males, the 7-day regimen of 60 mg base adult dose per day was well tolerated. It proved superior to the lower dose regimen in terms of late recurrences. As reinfection was excluded late recurrences could have resulted from either recrudescence or relapse. In previous studies in which primaquine was combined with artesunate, which has no radical curative activity but is highly effective against the blood stage infection,^5^,^7^,^8^ the recurrence rate with 7 days artesunate plus primaquine at a dose of 60 mg base/day was similar to that observed in the present study with primaquine only at 60 mg base/day. As recurrences following artesunate regimens are likely to be all relapses this suggests that the significantly higher recurrence rate (29% versus 7%) with the 30 mg/day primaquine monotherapy resulted both from the higher rate of relapses (~12%) but also a greater proportion of recrudescences (~17%) (Supplemental Appendix; Figure 4). This indicates that 30 mg/day does not provide maximum asexual stage activity whereas 60 mg/day provides 97% of maximal blood stage effect (Supplemental Appendix). The extrapolated dose-response relationship for relapse prevention (radical curative activity) suggests that 30 mg primaquine a day for 7 days gives approximately 72% of maximal relapse prevention, but is still inferior to the 60 mg/day regimen (92% of maximum) (Supplemental Appendix; Figure 4). These estimates are approximate as confidence intervals are wide, and it is possible that first recurrences (either relapse or recrudescence) could have occurred after the 28 day observation period. This confirms that primaquine has intrinsically weaker asexual stage activity than chloroquine or artemisinin derivatives,^13^ and it suggests that the dose response relationship for radical curative activity differs to that for asexual stage activity.

From a practical therapeutic perspective the higher dose shorter course regimen, if generally well tolerated, may be a preferable component of combination treatment regimens for vivax malaria. Further studies to assess tolerability in a broader age range are now indicated.

## Supplementary Material

Appendix: Estimation of Relapse and Recrudescence Rates in the Present Study

## Figures and Tables

**Table 1:** Demographic data and therapeutic responses in patients with *P. vivax* malaria in a comparison of 2 short-course primaquine-only regimens

Treatment groups	Primaquine 30 mg/day	Primaquine 60 mg/day	Both groups	*P* values
Numbers	43	42	85	
Age (years)	24.5 ± 9.9	24.6 ± 10.4	24.6 ± 10.1	0.94
Patients with previous malaria	17	20	37	0.59
No. of previous malaria infections median (range)	2 (1–10) *N* = 17	2 (1–7) *N* = 20	2 (1–10) *N* = 37	0.32
No. coming from the West of Thailand	38	36	74	0.99
History of fever (days)	4 (1–20)	4 (1–25)	4 (1–20)	0.93
Parasite count (/µL)	5,344 (33–97,968)	7,231 (445–73,476)	6,205 (33–97,968)	0.43
Hematocrit (%)	36.7 ± 5.9	35.8 ± 5.3	36.3 ± 5.6	0.50
White blood cell count (10^3^/µL)	6.9 ± 2.1	7.0 ± 2.1	7.0 ± 2.1	0.70

Data are shown as mean ± SD or median (range) and parasite count as geometric mean.

**Table 2:** Laboratory findings in patients with *P. vivax* malaria on admission and follow-up

Parameter	Primaquine 30 mg	Primaquine 60 mg	Both groups
Platelets (×10^9^/L) D0	111 ± 82; *N* = 41	112 ± 73; *N* = 41	111 ± 77; *N* = 82
Platelets (×10^9^/L) D7	288 ± 77; *N* = 38	274 ± 105; *N* = 37	281 ± 92; *N* = 75
Platelets (×10^9^/L) D14	281 ± 100; *N* = 34	285 ± 104; *N* = 37	283 ± 101; *N* = 71
Platelets (×10^9^/L) D28	232 ± 64; *N* = 27	227 ± 56; *N* = 32	229 ± 59; *N* = 59
Serum creatinine (mg/dL) D0	1.0 ± 0.2; *N* = 42	1.0 ± 0.3; *N* = 42	1.0 ± 0.2; *N* = 84
Serum creatinine (mg/dL) D7	0.9 ± 0.1; *N* = 39	0.9 ± 0.1; *N* = 38	0.9 ± 0.1; *N* = 77
Serum creatinine (mg/dL) D14	0.9 ± 0.1; *N* = 34	1.0 ± 0.1; *N* = 37	0.9 ± 0.1; *N* = 71
Serum creatinine (mg/dL) D28	0.9 ± 0.2; *N* = 27	0.9 ± 0.1; *N* = 32	0.9 ± 0.1; *N* = 59
Total bilirubin (mg/dL) D0	1.1 (0.4–5.4); *N* = 42	1.0 (0.1–4.1); *N* = 42	1.1 (0.1–5.4); *N* = 84
Total bilirubin (mg/dL) D7	0.6 (0.3–1.6); *N* = 39	0.6 (0.3–1.3); *N* = 38	0.6 (0.3–1.6); *N* = 77
Total bilirubin (mg/dL) D14	0.5 (0.2–1.4); *N* = 34	0.5 (0.3–1.2); *N* = 37	0.5 (0.2–1.4); *N* = 71
Total bilirubin (mg/dL) D28	0.5 (0.3–1.0); *N* = 27	0.4 (0.3–1.7); *N* = 32	0.5 (0.3–1.7); *N* = 59
SGPT (U/L) D0	24 (8–84); *N* = 42	19 (5–147); *N* = 42	21 (5–147); *N* = 84
SGPT (U/L) D7	32 (11–146); *N* = 39	24 (5–65); *N* = 38	27 (5–146); *N* = 77
SGPT (U/L) D14	35 (11–199); *N* = 34	20 (10–105); *N* = 37	26 (10–199); *N* = 71
SGPT (U/L) D28	22 (10–78); *N* = 27	18 (7–87); *N* = 32	19 (7–87); *N* = 59

D = Days after initiation of primaquine treatment; D0 = pretreatment values.

**Table 3:** Relationship between sexual and asexual parasitemia in patients with *P. vivax* gametocytemia

Parameters	Primaquine 30 mg	Primaquine 60 mg	Total	*P*
Gametocyte count/µL (median; range)				
Gametocytemia before treatment	36 (16–108) *N* = 15	55 (23–110) *N* = 10	41 (16–110) *N* = 25	0.06
Gametocytemia after treatment	46.5 (22–193) *N* = 14	55 (27–183) *N* = 14	49.5 (22–193) *N* = 28	0.61
All patients with gametocytemia	41 (16–193) *N* = 29	55 (23–183) *N* = 24	47 (16–193) *N* = 53	0.08
Gametocyte/parasite counts (%)				
Gametocytemia before treatment	0.4 (0.9–3.4) *N* = 15	0.6 (0.1–2.6) *N* = 10	0.5 (0.1–3.4) *N* = 25	0.69
Gametocytemia after treatment	0.8 (0.1–5.4) *N* = 14	0.6 (0.1–8.3) *N* = 14	0.6 (0.1–8.3) *N* = 28	0.64
All patients with gametocytemia	0.4 (0.1–5.4) *N* = 29	0.6 (0.1– 8.3) *N* = 24	0.6 (0.1–8.3) *N* = 53	0.44
Peak gametocyte/peak parasite counts (%)				
Gametocytemia before treatment	0.4 (0.1–3.4) *N* = 15	0.6 (0.1–2.6) *N* = 10	0.6 (0.1–3.4) *N* = 25	0.76
Gametocytemia after treatment	0.8 (0.1–4.0) *N* = 14	0.6 (0.2–2.4) *N* = 14	0.7 (0.1–4.0) *N* = 28	0.98
All patients with gametocytemia	0.4 (0.1–4.0) *N* = 29	0.6 (0.1–2.6) *N* = 24	0.6 (0.1–4.0) *N* = 53	0.79
Peak gametocyte/corresponding asexual parasite counts (%)				
Gametocytemia before treatment	0.9 (0.1–7.7) *N* = 15	0.6 (0.1–4.0) *N* = 10	0.7 (0.1–7.7) *N* = 25	0.80
Gametocytemia after treatment	3.0 (0.2–101) *N* = 14	0.6 (0.2–8.3) *N* = 14	1.0 (0.2–101) *N* = 28	0.016
All patients with gametocytemia	1.9 (0.1–101) *N* = 29	0.6 (0.1–8.3) *N* = 24	0.9 (0.1–101) *N* = 53	0.08

**Table 4:** Comparison between patients with and without patent *P. vivax* gametocytemia

No.	Patients with gametocytemia	Patients without gametocytemia	*P* values
	53	32	
Parasite count/μL (geometric mean; range)	8031; 522–97,968	4048; 33–49,738	0.06
Previous malaria attack (numbers)	0 (0–10)	0 (0–7)	0.80
Days of fever prior to admission	4 (1–15)	3.5 (1–20)	0.74
Hematocrit % (mean ± SD)	36.5 ± 5.5	35.8 ± 5.8	0.06
Fever clearance time (hours)	53.0 (4–201)	36.5 (4–94)	0.08
PCT50 (hours)	31.9 ± 17.9	22.6 ± 15.5	0.018
PCT90 (hours)	79.1 ± 21.7	67.0 ± 18.2	0.010
Parasite clearance time (hours)	94.4 ± 23.4	76.8 ± 25.0	0.002
No. of patients with *P. vivax* reappearance/FU patients	9/37 (24.3%)	2/27 (7.4%)	0.08

Data are shown as mean ± SD or median (range). FU = follow-up. PCT50 and PCT90 = times taken for the asexual parasite count fell by 50% and by 90% of the admission values.
