# Supplementary material for: A Comparison of Two Short-Course Primaquine Regimens for the Treatment and Radical Cure of Plasmodium vivax Malaria in Thailand
Source: Am J Trop Med Hyg. 2010 Apr;82(4):542–7. doi: 10.4269/ajtmh.2010.09-0428 (PMC2844579; doi:10.4269/ajtmh.2010.09-0428)
Supplement: Appendix: Estimation of Relapse and Recrudescence Rates in the Present Study [file SD1.pdf]

Am. J. Trop. Med. Hyg., 82(4), 2010, p. 1
doi:10.4269/ajtmh.2010.09-0428
Copyright © 2010 by The American Society of Tropical Medicine and Hygiene

APPENDIX: ESTIMATION OF RELAPSE AND RECRUDESCENCE RATES IN THE PRESENT STUDY

Data are taken from references 7 and 8 and the present study as in Figure 4. All calculations refer to 28 day assessments, and therefore do not take into account first recurrences after this time point. The estimates are approximate as numbers are relatively small and so confidence intervals for the proportions in each study are wide.

If the recurrence rate following treatment = R%, recrudescence rate = S%, and relapse = T%, then

$$R = S+T \qquad (1)$$

Assuming artesunate is completely curative for blood stage infection[8] (but has no hypnozoitocidal activity) then

$$R_{art} = T \qquad (2)$$

Thus any differences in recurrence rates between primaquine dosage regimens in artesunate- primaquine combination treatments result from differences in relapse rates (Figure 4 open circles).

Comparing recurrence rates with and without concomitant artesunate. For artesunate + 7 days primaquine$_{30}$ (total dose 210 mg) $R_{art} = T = 12\%$

For 7 days primaquine$_{30}$ (total dose 210 mg) without artesunate

$R_{30} = 29\%$, and as $T_{30} = 12\%$, and as from (1) $S = R - T$

$S_{30} = 17\%$

For artesunate + 7 days primaquine$_{60}$ (total dose 420 mg) $R_{art} = T = 4\%$

For 7 days primaquine$_{60}$ (total dose 420 mg) without artesunate

$R_{60} = 7\%$, and as $T_{60} = 4\%$,then as $S = R - T$
$S_{60} = 3\%$

In other words the difference in recrudescence rates between primaquine 30 mg/day for 7 days and 60 mg/day for 7 days without artesunate ($S_{60} - S_{30}$) is 17 – 3 = 14%

If this is correct then the 22% difference ($R_{30} - R_{60}$ = 29% – 7 % = 22%) without artesunate comprises approximately a 14% difference in recrudescence rates plus an 8% difference in relapse rates. This compares with an 8% difference between the 2 regimens with artesunate[7] attributable only to a difference in relapse rates.

Dose-response relationships. If maximum relapse prevention (i.e., T = 0) corresponds to prevention of 50% of recurrences[8] then

a) 7 days primaquine$_{60}$ provides (50 – 4/50) = 92% of the maximal hypnozoitocidal effect and
b) 7 days primaquine$_{30}$ provides (50 – 12/50) = 72% of the maximal hypnozoitocidal effect.

Assuming all infections could recrudesce, and censoring for those infections which relapse within 28 days, then

a) 7 days primaquine$_{60}$ alone provides (100 – 4) –3/(100 – 4) = 97% of maximal blood stage curative activity.
b) 7 days primaquine$_{30}$ alone provides (100 – 12) –17 /(100 – 12) = 81 % of maximal blood stage curative activity.
